# Effects of Asymmetric and Symmetric Sport Load on Upper and Lower Extremity Strength and Balance: A Comparison Between the Dominant and Non-Dominant Side in Adolescent Female Athletes

**DOI:** 10.3390/sports13030089

**Published:** 2025-03-14

**Authors:** Safoura Heshmati, Kourosh Ghahraman Tabrizi, Abdolhamid Daneshjoo, Elham Hosseini, Saeid Bahiraei, Mansour Sahebozamani, Andreas Konrad, David George Behm

**Affiliations:** 1 Department of Sports Injuries and Corrective Exercises, Faculty of Sports Sciences, Shahid Bahonar University of Kerman, Kerman 76169-14111, Iran; safoura.heshmat@sport.uk.ac.ir (S.H.); daneshjoo.hamid@uk.ac.ir (A.D.); hosseinielham7400@sport.uk.ac.ir (E.H.); s.bahiraei@uk.ac.ir (S.B.); sahebozamani@uk.ac.ir (M.S.); 2 Department of Sports Management, Faculty of Sports Sciences, Shahid Bahonar University of Kerman, Kerman 76169-14111, Iran; 3 School of Human Kinetics and Recreation, Memorial University of Newfoundland, St. John’s, NL A1C 5S7, Canada; dbehm@mun.ca; 4 Institute of Human Movement Science, Sport and Health, Graz University, Mozartgasse 14, 8010 Graz, Austria

**Keywords:** inter-limb asymmetry, limb dominance, strength, youth, unilateral imbalance

## Abstract

The aim of this research was to investigate the effects of primarily asymmetrical (soccer and volleyball) and symmetrical sport load (sprint and swimming) in the upper and lower limbs on dynamic balance and muscle strength and to compare these values in the dominant and non-dominant limbs. This study employed a cross-sectional design, included 45 adolescent female athletes from four sports, divided into asymmetric (ASYM, *n* = 25) and symmetric (SYM, *n* = 20) groups. They were assessed for maximal voluntary isometric muscle contraction (MVIC) relative muscular strength using a handheld dynamometer (HHD) for shoulder external rotation (ER) and internal rotation (IR), as well as hamstring and quadricep strength. Upper and lower limb balance were also assessed using the Upper (YBT-UQ) and Lower Quarter Y Balance Test (YBT-LQ) tests. The results showed significantly greater balance in the upper extremities of swimmers and in the lower extremities of the sprinters in both the dominant (DS) and non-dominant (NDS) sides than in other groups (*p* < 0.0001). However, no significant difference in internal and external shoulder rotator muscles strength between the groups (*p* > 0.05). Although significant differences were found in hamstring strength on the dominant side and quadricep strength on both sides (*p* < 0.05), a notable finding was that sprint athletes consistently demonstrated stronger quadriceps muscles as compared to other groups in both the dominant and non-dominant sides. According to the current findings, there are significant differences in upper and lower body balance, hamstring, and quadricep muscle strength among sports. This suggests that athletes of asymmetrical sports may need to improve non-dominant side knee strength and balance symmetry to prevent the risk of injury.

## 1. Introduction

Sports that primarily involve asymmetric execution of actions with the preferred limb, such as kicking in soccer or throwing in handball, may lead to the development of inter-limb asymmetries [1,2,3]. However, inter-limb asymmetries can also occur in symmetric sports that involve cyclic or alternating movement patterns, such as running, cycling, and swimming [4,5]. The literature suggests that morphological asymmetries (muscle strength, bone area, areal bone mineral content, and density) may be an adaptive consequence of long-term and intensive training in certain sports, such as year-round, intense training in a single sport [6,7,8]. Parrington et al. suggest that inter-limb asymmetries may arise from one limb being used more frequently than the other, due to uneven flexibility, range of motion, muscle strength development, and greater neural development on the preferred side [5]. Furthermore, inter-limb asymmetries indicate that one limb has lower function, physical capacity, and strength than the other [1,3].

Obviously, the number of repetitions of a particular movement type varies from sport to sport, influencing the possibility of asymmetry [6,9]. Failure to address asymmetry can lead to disproportionate changes in the volume and quality of muscle tissue and its contractile properties, thereby limiting the joint range of motion [6,10,11]. The degree of symmetry is determined by the training and activities specific to the sport. There are many examples of sports with significant levels of asymmetry, such as soccer and volleyball (in the lower extremities), and others without asymmetry, such as swimming, sprinting, and long-distance running [12,13]. According to a review of the literature, the limb preference of soccer players may result in strength imbalances that can significantly alter the myo-dynamic characteristics of the muscles of the dominant leg [3,14]. It has also been shown that asymmetries in strength are known to negatively affect the performance of sport-specific skills, such as kicking [15]. In particular, researchers found that because of the complex patterns of muscle activation required for joints’ stability, it is essential to assess and rehabilitate athletes by understanding the balance between agonist and antagonist muscle strength [16]. Asymmetries in inter-limb balance control are indicative of the lateralization of human movement control. The phenomenon under consideration could be attributed to hemispheric specialization for the analysis of somatosensory cues, with the non-dominant hemisphere (i.e., the right hemisphere for right-handed individuals) showing superior performance in somatosensory information processing [17]. Consequently, an increase in inter-limb balance asymmetries may have a detrimental effect on athletic performance and increase the likelihood of lower limb injuries, as they may result in uneven force distribution or a decrease in frontal plane stability, both of which are necessary to absorb the force of an impact [3,10].

In activities that are asymmetric and involve frequent shifts in the monopodal posture of the non-dominant leg to perform technical movements with the dominant leg (DL) (such as passing and kicking in soccer), the non-dominant leg (NDL) may exhibit greater postural control as compared to the dominant leg [18]. The NDL is typically used to maintain balance during a single-legged stance, whereas the DL is typically used to perform complex lower limb movements such as kicking or tracing shapes [17]. Whilst some studies have reported differences in postural control between the DLs and NDLs of athletes, other research has shown similar postural control between the DLs and NDLs in different groups of athletes [18,19,20]. Based on the present findings, only two studies have compared monopodal postural control in the DLs and NDLs of expert athletes participating in asymmetric and symmetric sports, so this theory remains to be proven [18]. This high level of imbalance creates a significant risk of injury during training and competition, as well as in everyday activities [12]. Therefore, monitoring and improving muscle strength imbalances may lead to improved performance and reduced risk of injury [3,10].

Both limbs may be at greater risk of injury due to asymmetry. Athletes may find it difficult to withstand even normal strength on the weaker leg, whereas the stronger leg may be able to withstand very high strength due to increased dependence and high loading on that side. Bilateral lower limb asymmetry has been reported in lateral and non-lateral dominant sports [21]. In addition, a recent study found that bilateral asymmetry in sport may be normal and unrelated to poor performance or injury [22]. However, Svensson et al. [23] analyzed potential differences in muscle injury type, localization, and severity between the dominant and non-dominant legs of male soccer players. They found that more severe structural hamstrings injuries occurred in the dominant legs as compared to the non-dominant legs. In addition, studies have shown that athletes with significant strength asymmetries are several times more likely to suffer muscle injuries that could affect performance (such as a hamstring strain) than athletes without asymmetries [24]. In addition, motor control theory suggests that asymmetry may limit an athlete’s movement strategies. Therefore, athletes may develop motor behaviors that increase their risk of injury [25]. In general, the lack of agreement on the effects of limb dominance may be due to the nature of the sport. For example, due to their highly specialized and varied demands, team sports such as soccer may increase lower limb asymmetries [24], and volleyball may increase upper limb asymmetries. To gain a comprehensive understanding of how asymmetry naturally emerges in different sports, an in-depth investigation is necessary. This study is designed to examine the differences in muscle strength and dynamic balance among athletes engaged in asymmetric versus symmetric sports, with a particular emphasis on the role of limb dominance. Our goal is to elucidate how the specific characteristics of athletic activities contribute to the development of asymmetry in physical capabilities. It was hypothesized that athletes participating in primarily asymmetric sports would exhibit greater muscle strength and dynamic balance in their dominant limbs as compared to their non-dominant limbs. Conversely, athletes participating in symmetric sports were expected to show no significant differences in muscle strength or dynamic balance between their dominant and non-dominant limbs. Furthermore, it was hypothesized that there would be a significant interaction effect between the type of sport (asymmetric vs. symmetric) and limb dominance (dominant vs. non-dominant) on both muscle strength and dynamic balance.

## 2. Materials and Methods

### 2.1. Study Design and Participants

**Figure 1:**
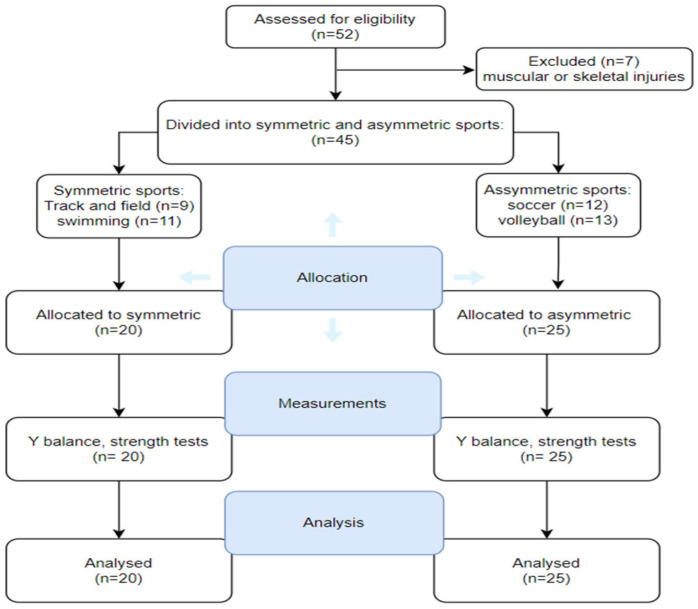
Flow chart.

This cross-sectional study was carried out from June to August 2023, involving female athletes from four different club sports in Kerman. Initially, 52 participants were enrolled in this study, but this number was adjusted to 45 after applying the inclusion and exclusion criteria. The participants were categorized into two groups: those engaged in primarily asymmetric sports (ASYM, *n* = 25) and those involved in symmetric sports (SYM, *n* = 20), based on the nature of the movements performed during their athletic activities, specifically whether the movements of the right and left sides were symmetric or asymmetric (Figure 1). This study adhered to a conventional academic structure and employed clear, objective language with precise word choice. All participants had at least three years of training experience and regular training at least three times per week. Demographic characteristics and sports participation details for both ASYM and SYM groups are presented in Table 1. The sample size was estimated by using G * Power software (Version 3.1.9.4) (one way ANOVA, α = 0.05, ES = 0.50) with a statistical power of 0.8 and literature review [3,6,10,13].

Inclusion criteria included athletes under the age of 18 with at least three years of regular training experience in the relevant sport (three sessions each week). Participants with muscular or skeletal injuries were excluded. Participants were instructed to avoid performing physically demanding activities or consuming stimulating substances such as caffeinated beverages within 24 h of the data collection. All athletes followed a similar lifestyle and were observed by one of the researchers during regular training. Two sports science specialists evaluated the athletes using a blind process that followed the same standards.

### 2.2. Procedures

The first step involved taking anthropometric measurements, such as height, arm and leg length, and body weight. To determine limb dominance, participants were asked to either kick a soccer ball or throw a ball, and the limb used for ball-kicking was considered the dominant limb [26]. Each of the four sports, which included sprint sports, swimming, volleyball, and soccer (in both symmetric and asymmetric groups), underwent separate training seasons. The athletes trained at least three times per week for their sports. Each session lasted about one and a half to two hours.

Participants traveled short distances of 5–10 min by car to the test location. All participants were instructed to maintain their regular sleep routines. Participants completed relative strength and balance assessments in one session. During data collection, participants wore their sport’s shoes and clothing. A standard warm-up, including dynamic stretching of the lower and upper extremities’ muscles, was conducted before each session (10 min) [27]. Tests were randomly performed on both the dominant and non-dominant sides. The evaluations were conducted by sports science specialists E.H. and S.H. in a laboratory setting of the Shahid Bahonar University of Kerman. The relative strength of their lower limbs was assessed using a handheld dynamometer (HHD), Relative muscle strength assessment normalizes strength measurements by dividing an individual’s muscle strength data by their body weight, facilitating equitable comparisons and progress monitoring across individuals. The strength of their lower limbs was assessed using a handheld dynamometer (HHD), while their upper limb balance was evaluated using the upper-quarter Y balance test (YBT–UQ). Additionally, their lower limb balance was measured using the lower-quarter Y balance test (YBT–LQ) [28]. Every subject experienced the same lighting, temperature, and noise levels throughout the testing procedure. All participants were advised to maintain the same eating and sleeping routines. The testing procedures took about 30 min to complete. Each testing measurement was performed between 8 a.m. and 11 a.m.

### 2.3. Lower Quarter Y Balance Test (YBT-LQ)

The YBT–LQ was assessed using the YBT Kit (Functional Movement Systems^®^) (ICC = 0.99) [29]. The kit consists of three pipes connected to a central platform, which represent the anterior (AT), posteromedial (PM), and posterolateral (PL) reach directions. Each pipe has a movable reach indicator, marked with 1.0 cm intervals for accurate measurement. Each participants was instructed to stand on the central platform with their left leg and push the reach indicator as far as possible in the AT direction. They were then instructed to switch to their right leg and perform for the PM and PL directions. Each participant completed three trials for each leg and reach direction following three practice trials. A one-minute break was given between each trial. This was calculated by dividing the reach distance by the lower limb length and multiplying by 100 [28].

### 2.4. Upper Quarter Y Balance Test (YBT-UQ)

The YBT Kit was used to assess YBT–UQ test (ICC = 0.90) by instructing each participant to extend the reach indicator as far as possible in the medial (MD), inferolateral (IL), and superolateral (SL) directions with their right arm while maintaining a weight-bearing one-arm push-up posture with their left arm on the center platform [29]. The same procedure was replicated with the left arm. Each participant completed three practice trials followed by three data-gathering trials, with a one-minute interval between attempts. The best values, specifically the absolute maximum reach distance in centimeters, were recorded for each arm and reach direction for further analysis. In young, healthy individuals, the average reach distance was expressed as a percentage of upper limb length. This was calculated by dividing the reach distance by the upper limb length and multiplying by 100 [28].

### 2.5. Shoulder External Rotation and Internal Rotation Strength

Shoulder external rotation (ER) and internal rotation (IR) strengths were assessed using a HHD with a 0–500 N range and a 0.2 N sensitivity (Nicholas Manual Muscle Test, Co., Lafayette, IN, USA). The HHD was calibrated prior to each test based on the manufacturer’s instructions. Participants assumed a supine posture on a bench, with their arms abducted at 90° and rotated at 0° in the scapular plane. Each participant’s humerus was placed against the bench, and their elbow was flexed to a 90° angle to confirm the testing angle visually [30]. To evaluate ER strength, the participants rotated their shoulders outward against the HHD, which was positioned near the ulnar styloid process. For IR strength, the participants rotated their shoulders inward against the HHD, which was positioned close to the radius styloid process. The HHD was securely fixed to a flat and sturdy framework to ensure stability [31]. Each participant completed three sets of maximum voluntary isometric contractions (MVIC), for 5 s each for both the ER and IR tests. There was a 2-min rest interval between sets. Peak strength was recorded for each of the three repetitions and normalized to body mass in kilograms. The maximal isometric muscle strength of the shoulder lateral and medial rotators were assessed using the calibrated HHD [30]. This device has demonstrated excellent inter-rater reliability (intraclass correlation coefficient ICC = 0.67 to 0.99) and intra-rater reliability (ICC = 0.67 to 0.96) in tests of isometric muscular strength [31].

### 2.6. Hamstrings and Quadriceps Strength

The MVIC of the hamstrings and quadriceps was assessed using a HHD fixed bench. To minimize bias, the examined muscle was randomly assigned. The athlete was evaluated in a seated position with their hips and knees bent at a 90-degree angle, and the MVIC strengths of their quadriceps and hamstrings were tested. The subject’s arms were secured to their chest, and their legs were fastened to the stretcher as close to the hips as possible. They were instructed to maintain a vertical body posture. The strength of their hamstrings was assessed using the push method, positioning the HHD at the farthest distal point on the back of the leg. To test the strength of their quadriceps, the pull technique was applied with a band positioned perpendicular to the leg, 5 cm above the lateral malleolus. The dynamometer was firmly fixed to the stretcher using screws. Strength assessments were normalized to body weight to control for inter-individual variability and minimize measurement error [32,33,34].

### 2.7. Statistical Analyses

Statistical analysis was conducted using SPSS Version 26 (Armonk, NY, USA: IBM Corp.). The homogeneity of variance among conditions and the normality of the distribution of scores were assessed using Levene’s and Shapiro–Wilk’s tests, respectively (*p* > 0.05). Dependent variables among the groups were compared using two one-way analyses of variance (ANOVA). The post-hoc Scheffé test was conducted to identify pairwise differences when main effect differences were observed. The effect size was assessed using partial eta squared and classified as small (pη^2^ = 0.01), medium (pη^2^ = 0.06), or large (pη^2^ = 0.14). Additionally, Cohen’s d was used to test the effect sizes of two independent groups: d = (M1 − M2/√ ((SD1^2^ × SD2^2^)/2), with 0.2, 0.5, and 0.8 considered as small-, medium-, and large-magnitude effects, respectively [35]. A significance level of *p*-value ≤ 0.05 was accepted for all statistical parameters.

## 3. Results

### 3.1. Balance

#### 3.1.1. Upper Quarter Y Balance

**Figure 2:**
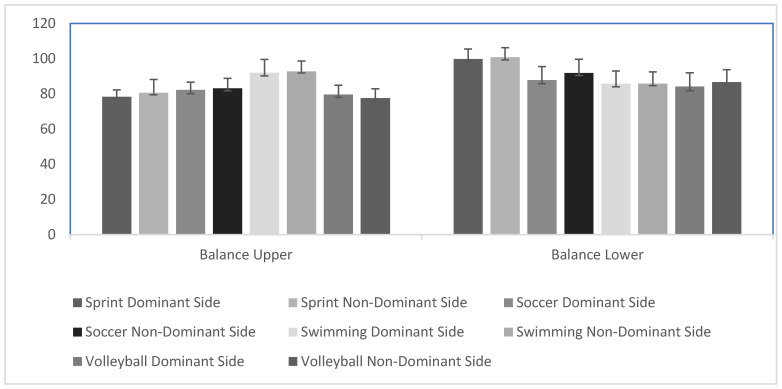
Balance among groups. The Y axis depicts balance (cm) values.

Analysis of variance (ANOVA) revealed significant differences among groups in upper extremity balance for both the dominant sides (DSs) (F3,41 = 13.35, *p* < 0.0001, pη^2^ = 0.49) and non-dominant sides (NDSs) (F3,41 = 13.25, *p* < 0.0001, pη^2^ = 0.49). Post-hoc tests indicated that swimmers had significantly greater balance than sprinters (DS: *p* < 0.0001, effect size = 2.25; NDS: *p* = 0.001, effect size = 1.79), soccer players (DS: *p* = 0.002, effect size = 1.55; NDS: *p* = 0.006, effect size = 1.65), and volleyball players (DS: *p* < 0.0001, effect size = 1.87; NDS: *p* < 0.0001, effect size = 2.71). No significant differences were found among sprinters, soccer players, and volleyball players for upper extremity balance (*p* > 0.05) (Table 2, Figure 2).

#### 3.1.2. Lower Quarter Y Balance

For balance in the lower extremities, ANOVA showed significant differences among groups in both DSs (F3,41 = 9.69, *p* < 0.0001, pη^2^ = 0.41) and NDSs (F3,41 = 9.74, *p* < 0.0001, pη^2^ = 0.42). Sprinters demonstrated significantly greater balance as compared to soccer players (DS: *p* = 0.006, effect size = 1.78; NDS: *p* = 0.049, effect size = 1.34), swimmers (DS: *p* = 0.001, effect size = 2.19; NDS: *p* < 0.0001, effect size = 2.46), and volleyball players (DS: *p* < 0.0001, effect size = 2.31; NDS: *p* < 0.0001, effect size = 2.25). No significant differences were observed among soccer players, swimmers, and volleyball players for lower extremity balance (*p* > 0.05) (Table 2, Figure 2).

### 3.2. Strength

#### 3.2.1. Strength of Upper Extremities

The analysis revealed no significant differences among groups for the strength of shoulder internal rotators for both the dominant sides (DSs) and non-dominant sides (NDSs). Specifically, the results for the DSs showed (F3,41 = 1.20, *p* = 0.28), and for the NDSs, the results showed (F3,41 = 2.49, *p* = 0.07). Similarly, no significant differences were observed in the strength of the external rotators for the DSs (F3,41 = 0.67, *p* = 0.57) and the NDSs (F (3,41) = 1.81, *p* = 0.15). Post-hoc tests further confirmed that no significant differences existed between groups (*p* > 0.05).

#### 3.2.2. Strength of Lower Extremities

**Figure 3:**
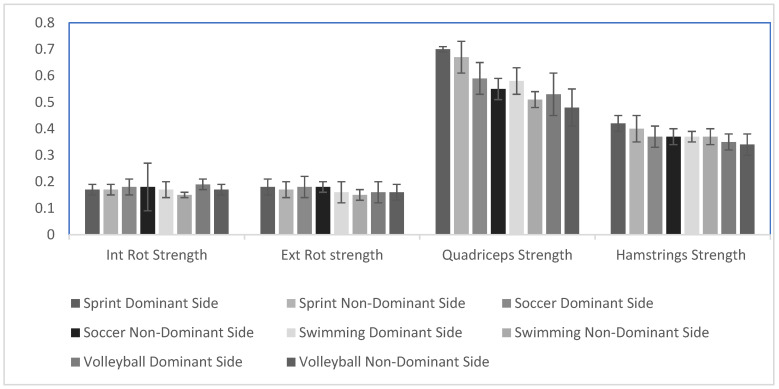
Relative muscle strength among groups. The Y axis depicts relative strength (kilograms/body mass) values.

Significant differences were observed among the groups for hamstrings strength for DSs (F3,41 = 2.79, *p* = 0.02); however, no significant differences in NDSs (F3,41 = 5.76, *p* = 0.05) were found. Post-hoc tests indicated that sprinters exhibited greater hamstring strength as compared to soccer and volleyball players on the DSs (*p* = 0.04), (*p* = 0.001). Additionally, sprinters showed higher hamstring strength than volleyball players on the NDSs (*p* = 0.03). No significant differences were found among other groups (*p* > 0.05). For quadricep strength, significant differences were noted in both DSs (F3,41 = 8.49, *p* = 0.001) and NDSs (F3,41 = 20.18, *p* = 0.001). Specifically, sprinters demonstrated higher quadricep strength than soccer, swimmers, and volleyball players on the DSs: (*p* = 0.01), (*p* = 0.01), and (*p* = 0.001), respectively. On the NDSs, sprinters had greater quadriceps strength than volleyball, soccer, and swimmers (*p* = 0.001 for all) Furthermore, a significant difference was noted between soccer and volleyball players on the NDSs (*p* = 0.02). No other significant differences were observed (*p* > 0.05), (Table 3, Figure 3).

## 4. Discussion

The hypothesis of this study was that athletes participating in asymmetric sports would exhibit greater muscle strength and dynamic balance in their dominant limbs as compared to their non-dominant limbs. Conversely, athletes participating in symmetric sports were expected to show no significant differences in muscle strength or dynamic balance between their dominant and non-dominant limbs. Furthermore, it was hypothesized that there would be a significant interaction effect between the type of sport (asymmetric vs. symmetric) and limb dominance (dominant vs. non-dominant) on both muscle strength and dynamic balance. The current findings revealed significant differences in balance between the upper and lower body in the different sports of soccer, volleyball, sprint, and swimming. Compared to the other sports, female swimmers (symmetry sport) showed significantly better outcomes in upper limb dynamic balance, while sprinters (symmetry sport) demonstrated significantly better results in lower limb dynamic balance in both dominant and non-dominant limbs. According to Bartolomeu et al., a higher score in dynamic balance improves swimming technique [36]. These results suggest that the demands of YBT–LQ and YBT–UQ are related to the athletic requirements [28]. However, a closer examination of the results shows that the differences between limbs in this investigation are lower than in previous studies [24,37,38]. Plisky et al. discovered that individuals with a left/right imbalance greater than 4 cm on the YBT were 2.5 times more likely to have experienced a lower limb injury. Based on the prior related literature [37,38,39], these findings demonstrate that comparing limbs can be a useful and quick screening technique for assessing the risk of lower limb injury. Furthermore, research in this field has demonstrated that asymmetry in the lower limbs heightens the likelihood of injury and affects the performance of players [40,41].

To comprehend the current results, two theories are referenced. The first theory suggests that female athletes may be affected by muscular imbalances and changes in tissue stress due to the high volume of repeated asymmetric movement patterns present in asymmetric sports [42,43]. In this regard, studies have revealed a correlation between a lower extremity injury risk and asymmetry to maintain the dynamic balance ability in a variety of populations [39,44]. All athletes who have considerable reach asymmetry in the YBT are susceptible to experiencing non-contact injuries [24,38]. Iga et al. reported that players rarely use both legs equally because their preference for one side over the other is related to the brain’s hemisphere dominance on the opposite side [45]. This fact may influence certain preferences, leading to morphological and pathophysiological differences in athletes who favor one side over the other [6,45]. However, according to the second theory, unilateral periodic activities such as hitting the ball, jumping, and landing are less impacted by dominating bilateral activities like sprinting and running. This is supported by the minimal observed difference. Bilateral activities help to balance the natural tendency to favor the dominant limb when landing and hitting the ball, as sprinting, agility, and passing all require symmetrical activities [24]. Also it is important to note that the dynamic balance factor depends on various factors, including range of motion, sex, motor skills, strength, and proprioception [46].

However, there was no significant difference in the strength of the internal rotator and external rotator muscles among sports and the dominant and non-dominant limbs. These results are not consistent with previous research indicating that female volleyball players have greater IR strength in their dominant shoulders than in their non-dominant shoulders [47,48,49]. Differences in IR and ER strength ratios are linked to injury in athletes participating in overhead throwing activities. As previously mentioned, IR concentric strength correlates well with athletes’ performances [47]. Research on volleyball and handball players indicates that, although the dominant arm can show an increase in strength as a result of repeated use, these variations are frequently not statistically significant in controlled testing environments such as isometric dynamometry [47]. Additionally, the mechanics of the motion of overhead or throwing activities requires matching movements of the complete kinetic chain, reducing dependence on unilateral shoulder dominance. Similar strength adaptations are promoted over time by the non-dominant arm’s stabilizing or extra help function, even in asymmetrical sports [48]. Furthermore, younger athletes’ musculoskeletal systems are still developing; hence, they might not show obvious asymmetries. These results indicate that at this developmental period, significant variations in shoulder internal rotator strength are limited [49].

Also, there was a significant difference in the hamstrings of dominant limbs and in quadricep strength among the sports groups in dominant and non-dominant limbs. Specifically, runners demonstrated greater strength in their hamstrings and quadriceps. It has been shown in earlier research that athletes, especially sprinters, who possess stronger knee extensor muscles perform better in sprints [50]. Running economy (RE) is a critical factor in endurance performance and is influenced by muscle strength balance, particularly between the hamstrings and quadriceps [51]. Research indicates that resistance training (RT), which includes strength and plyometric exercises, enhances neuromuscular efficiency, tendon stiffness, and muscle–tendon unit properties [52]. Balanced strength in the hamstrings and quadriceps optimizes joint stability and stride mechanics, lowering metabolic energy costs during running [51]. Eccentric muscle strength and quasi-stiffness are also positively correlated with running economy, as they allow better utilization of elastic energy during the stretch-shortening cycle [52].

Most of the athletes showed less than 10% strength and balance asymmetry, which supports the literature that advocates that less than 10% asymmetry is a criterion for returning to sports [53,54,55]. A recent systematic evaluation revealed that strength asymmetry between limbs differed among groups and included findings indicating asymmetry of greater than 15%. Additionally, there were no discernible connections between the asymmetries and independent performance tasks, such as seated shot put, isokinetic dynamometry, and jump tests [56]. Although asymmetric motions and unilateral actions are common in many sports, they do not always lead to inter-limb asymmetry. On the other hand, sprinters and swimmers showed less asymmetry between the dominant and non-dominant limbs due to the nature of the sport. The findings of this study have significant implications for fitness trainers, physiotherapists, doctors, and other clinical professionals. To enhance the practical application of these findings, it is recommended that training programs incorporate strategies aimed at injury prevention and improving balance symmetry or strengthening weaker sides, especially in athletes participating in asymmetrical sports. For instance, coaches could implement targeted exercises that focus on the non-dominant limb to foster balance and strength improvements.

While this study provides valuable insights, it is important to acknowledge several limitations that may affect the interpretation of the findings. First, the sample size of 45 female athletes is relatively small. This limitation restricts the generalizability of our results to a broader population. Future research should aim to include a larger and more diverse sample to validate these findings and enhance their applicability. Additionally, this study focused on symmetry and asymmetry in sports fields, yet few studies have explored the impact of symmetric versus asymmetric exercises on dynamic balance and muscle strength in either the upper or lower limbs. This gap in the literature highlights an area for further investigation, as understanding these effects could provide deeper insights into training and rehabilitation strategies. Another notable limitation is the exclusion of prominent asymmetrical sports such as badminton, tennis, and fencing. These activities involve specific movement patterns and equipment (e.g., racquets and swords) that may influence muscle strength and balance differently than the sports included in this study. Including athletes from these sports in future research could yield valuable comparative data on asymmetry. Furthermore, while participants were categorized based on four different sports, we did not account for potential differences and asymmetries within each sport based on an athlete’s specialty or role. For example, a swimmer’s technique or a volleyball player’s position may lead to varying degrees of muscle strength asymmetry. Future studies should consider these intra-sport variations to provide a more comprehensive understanding of how specialization affects muscle dynamics. In summary, addressing these limitations in future research will not only strengthen the validity of our findings but also contribute to a more nuanced understanding of muscle strength asymmetries across different sports contexts. This revision expands on each limitation, justifies its significance, and suggests directions for future research while connecting the discussion to the existing literature.

## 5. Conclusions

This research confirms that female athletes in asymmetric sports exhibit greater dynamic balance in their dominant limbs as compared to their non-dominant limbs, while those in symmetric sports show no significant differences. Key findings highlight the fact that female swimmers excel in upper limb dynamic balance and sprinters achieve superior lower limb balance, illustrating sport-specific adaptations. This study also indicated that track and field players had stronger hamstring and quadriceps strength in their dominant and non-dominant limbs as compared to other sports. This study emphasizes the importance of targeted training for balance and strength, particularly for the non-dominant limb, to reduce injury risk and enhance performance among females in asymmetric sports.

## Figures and Tables

**Table 1:** Participants’ demographic characteristics (values are mean ± SD).

	ASYM	SYM	*p*-Value
Age (years)	16.68 ± 0.80	16.35 ± 1.18	0.12
Height (cm)	163.92 ± 6.38	163.95 ± 4.67	0.13
Body weight (kg)	53.64 ± 6.03	52.30 ± 5.76	0.67
BMI (kg·m^2^)	19.94 ± 1.67	19.44 ± 1.81	0.50
Sport	Soccer (*n* = 12)	Sprint (*n* = 9)	-
	Volleyball (*n* = 13)	Swimming (*n* = 11)	-

SYM: symmetry sports; ASYM: asymmetry sports; *p*-value = *p*-value of independent sample *t*-test between groups.

**Table 2:** Balance among groups (values are mean centimeters ± SD).

Balance UE						
Group	Dominant	Sig	Effect Size	Non-Dominant	Sig	Effect Size
Sprint	78.3 ± 3.9	0.001	0.494	80.6 ± 7.5	0.001	0.492
Soccer	82.2 ± 4.5			83.1 ± 5.7		
Swimming	91.9 ± 7.6 ^a,b,c,d^			92.7 ± 5.9 ^a,b,c,d^		
Volleyball	79.6 ± 5.3			77.5 ± 5.3		
**Balance LE**						
Group	Dominant	Sig	Effect Size	Non-Dominant	Sig	Effect Size
Sprint	99.8 ± 5.6 ^a,b,c,d^	0.001	0.415	100.8 ± 5.4 ^a,b,c,d^	0.001	0.416
Soccer	87.8 ± 7.7			91.8 ± 7.8		
Swimming	85.8 ± 7.1			85.7 ± 6.8		
Volleyball	84.1 ± 7.8			86.6 ± 7.1		

Note: UE = upper extremities, LE = lower extremities, a = significant difference with sprint group (*p* < 0.05), b = significant difference with soccer group (*p* < 0.05), c = significant difference with swimming group (*p* < 0.05), d = significant difference with volleyball group (*p* < 0.05).

**Table 3:** Relative muscles strength among groups (values are mean ± SD) (kilograms/body mass).

	Group	Dominant	Sig	Non-Dominant	Sig
Strength (Internal Rotators)	Sprint	0.17 ± 0.20	0.31	0.17 ± 0.02	0.074
	Soccer	0.18 ± 0.03		0.18 ± 0.09	
	Swimming	0.17 ± 0.03		0.15 ± 0.01	
	Volleyball	0.19 ± 0.02		0.17 ± 0.02	
Strength (External Rotators)	Sprint	0.18 ± 0.03	0.57	0.17 ± 0.03	0.15
	Soccer	0.18 ± 0.04		0.18 ± 0.02	
	Swimming	0.16 ± 0.04		0.15 ± 0.02	
	Volleyball	0.16 ± 0.04		0.16 ± 0.03	
MVIC (Quadriceps Strength)	Sprint	0.70 ± 0.01 ^b,c,d^	0.001	0.67 ± 0.06 ^b,c,d^	0.001
	Soccer	0.59 ± 0.06 ^a^		0.55 ± 0.04 ^a,d^	
	Swimming	0.58 ± 0.05 ^a^		0.51 ± 0.03 ^a^	
	Volleyball	0.53 ± 0.08 ^a^		0.48 ± 0.07 ^a^	
MVIC (Hamstrings Strength)	Sprint	0.42 ± 0.03 ^b,d^	0.002	0.40 ± 0.05 ^d^	0.052
	Soccer	0.37 ± 0.04 ^a^		0.37 ± 0.03	
	Swimming	0.37 ± 0.02		0.37 ± 0.03	
	Volleyball	0.35 ± 0.03 ^a^		0.34 ± 0.04 ^a^	

Note: MVIC: maximal voluntary isometric contraction, a = significant difference with sprint group (*p* < 0.05), b = significant difference with soccer group (*p* < 0.05), c = significant difference with swimming group (*p* < 0.05), d = significant difference with volleyball group (*p* < 0.05).

## Data Availability

The data presented in this study are available on request from the corresponding author due to the privacy of the participants.

## References

[1] Heil J., Loffing F., Büsch D. (2020). The influence of exercise-induced fatigue on inter-limb asymmetries: A systematic review. Sports Med. Open.

[2] Bromley T., Turner A., Read P., Lake J., Maloney S., Chavda S., Bishop C. (2021). Effects of a competitive soccer match on jump performance and interlimb asymmetries in elite academy soccer players. J. Strength Cond. Res..

[3] Fox K.T., Pearson L.T., Hicks K.M. (2023). The effect of lower inter-limb asymmetries on athletic performance: A systematic review and meta-analysis. PLoS ONE.

[4] Maloney S.J. (2019). The relationship between asymmetry and athletic performance: A critical review. J. Strength Cond. Res..

[5] Parrington L., Ball K. (2016). Biomechanical considerations of laterality in sport. Laterality in Sports.

[6] Kalata M., Maly T., Hank M., Michalek J., Bujnovsky D., Kunzmann E., Zahalka F. (2020). Unilateral and bilateral strength asymmetry among young elite athletes of various sports. Medicina.

[7] Hart N.H., Nimphius S., Weber J., Spiteri T., Rantalainen T., Dobbin M., Newton R. (2016). Musculoskeletal asymmetry in football athletes: A product of limb function over time. Med. Sci. Sports Exerc..

[8] Brenner J.S., LaBella C.R., Brookes M.A., Diamond A., Hennrikus W., Kelly A.K.W., LaBotz M., Logan K., Loud K.J., Moffatt K.A. (2016). Sports specialization and intensive training in young athletes. Pediatrics.

[9] Pion J., Lenoir M., Vandorpe B., Segers V. (2015). Talent in female gymnastics: A survival analysis based upon performance characteristics. Int. J. Sports Med..

[10] Ličen U., Kozinc Ž. (2023). The influence of inter-limb asymmetries in muscle strength and power on athletic performance: A review. Monten. J. Sports Sci. Med..

[11] Maly T., Sugimoto D., Izovska J., Zahalka F., Mala L. (2018). Effect of muscular strength, asymmetries and fatigue on kicking performance in soccer players. Int. J. Sports Med..

[12] Bartol V., Vauhnik R., Rugelj D. (2022). Influence of the sport specific training background on the symmetry of the single legged vertical counter movement jump among female ballet dancers and volleyball players. Heliyon.

[13] Vaisman A., Guiloff R., Rojas J., Delgado I., Figueroa D., Calvo R. (2017). Lower limb symmetry: Comparison of muscular power between dominant and nondominant legs in healthy young adults associated with single-leg-dominant sports. Orthop. J. Sports Med..

[14] Fousekis K., Tsepis E., Vagenas G. (2010). Lower limb strength in professional soccer players: Profile, asymmetry, and training age. J. Sports Sci. Med..

[15] Bishop C., Turner A., Read P. (2018). Effects of inter-limb asymmetries on physical and sports performance: A systematic review. J. Sports Sci..

[16] Leahy I., Florkiewicz E., Shotwell M.P. (2024). Isokinetic Dynamometry for External and Internal Rotation Shoulder Strength in Youth Athletes: A Scoping Review. Int. J. Sports Phys. Ther..

[17] Brighenti A., Noé F., Stella F., Schena F., Mourot L. (2022). Warm-Up Improves Balance Control Differently in the Dominant and Non-Dominant Leg in Young Sportsmen According to Their Experience in Asymmetric or Symmetric Sports. Int. J. Environ. Res. Public Health.

[18] Kadri M.A., Noé F., Maitre J., Maffulli N., Paillard T. (2021). Effects of limb dominance on postural balance in sportsmen practicing symmetric and asymmetric sports: A pilot study. Symmetry.

[19] Sabin M.J., Ebersole K.T., Martindale A.R., Price J.W., Broglio S.P. (2010). Balance performance in male and female collegiate basketball athletes: Influence of testing surface. J. Strength Cond. Res..

[20] Matsuda S., Demura S., Demura T. (2010). Examining differences between center of pressure sway in one-legged and two-legged stances for soccer players and typical adults. Percept. Mot. Skills.

[21] Guan Y., Bredin S., Taunton J., Jiang Q., Wu L., Kaufman K., Wu N., Warburton D. (2021). Bilateral difference between lower limbs in children practicing laterally dominant vs. non-laterally dominant sports. Eur. J. Sport Sci..

[22] Bonavolontà V., Gallotta M.C., Zimatore G., Curzi D., Ferrari D., Vinciguerra M.G., Guidetti L., Baldari C. (2023). Chronic Effects of Asymmetric and Symmetric Sport Load in Varsity Athletes across a Six Month Sport Season. Int. J. Environ. Res. Public Health.

[23] Svensson K., Eckerman M., Alricsson M., Magounakis T., Werner S. (2018). Muscle injuries of the dominant or non-dominant leg in male football players at elite level. Knee Surg. Sports Traumatol. Arthrosc..

[24] Haddad M., Abbes Z., Zarrouk N., Aganovic Z., Hulweh A., Moussa-Chamari I., Behm D.G. (2023). Difference Asymmetry between Preferred Dominant and Non-Dominant Legs in Muscular Power and Balance among Sub-Elite Soccer Players in Qatar. Symmetry.

[25] Helme M., Tee J., Emmonds S., Low C. (2021). Does lower-limb asymmetry increase injury risk in sport? A systematic review. Phys. Ther. Sport.

[26] Hosseini E., Daneshjoo A., Sahebozamani M., Behm D. (2021). The effects of fatigue on knee kinematics during unanticipated change of direction in adolescent girl athletes: A comparison between dominant and non-dominant legs. Sports Biomech..

[27] Henriques-Neto D., Minderico C., Peralta M., Marques A., Sardinha L.B. (2020). Test–retest reliability of physical fitness tests among young athletes: The FITescola^®^ battery. Clin. Physiol. Funct. Imaging.

[28] Schwiertz G., Beurskens R., Muehlbauer T. (2020). Discriminative validity of the lower and upper quarter Y balance test performance: A comparison between healthy trained and untrained youth. BMC Sports Sci. Med. Rehabil..

[29] Ruffe N.J., Sorce S.R., Rosenthal M.D., Rauh M.J. (2019). Lower quarter-and upper quarter Y balance tests as predictors of running-related injuries in high school cross-country runners. Int. J. Sports Phys. Ther..

[30] Hams A.H., Evans K., Adams R., Waddington G., Witchalls J. (2019). Shoulder internal and external rotation strength and prediction of subsequent injury in water-polo players. Scand. J. Med. Sci. Sports.

[31] Beshara P., Davidson I., Pelletier M., Walsh W.R. (2022). The Intra-and Inter-Rater Reliability of a Variety of Testing Methods to Measure Shoulder Range of Motion, Hand-behind-Back and External Rotation Strength in Healthy Participants. Int. J. Environ. Res. Public Health.

[32] Reurink G., Goudswaard G.J., Moen M.H., Tol J.L., Verhaar J.A., Weir A. (2016). Strength measurements in acute hamstring injuries: Intertester reliability and prognostic value of handheld dynamometry. J. Orthop. Sports Phys. Ther..

[33] Whiteley R., Jacobsen P., Prior S., Skazalski C., Otten R., Johnson A. (2012). Correlation of isokinetic and novel hand-held dynamometry measures of knee flexion and extension strength testing. J. Sci. Med. Sport.

[34] Lipovšek T., Kacin A., Puh U. (2022). Reliability and validity of hand-held dynamometry for assessing lower limb muscle strength. Isokinet. Exerc. Sci..

[35] Pallant J. (2020). SPSS Survival Manual: A Step by Step Guide to Data Analysis Using IBM SPSS.

[36] Bartolomeu R.F., Sampaio T., Oliveira J.P., Barbosa T.M., Morais J.E. (2023). Association between the upper quarter dynamic balance, anthropometrics, kinematics, and swimming speed. J. Funct. Morphol. Kinesiol..

[37] Gorman P.P., Butler R.J., Rauh M.J., Kiesel K., Plisky P.J. (2012). Differences in dynamic balance scores in one sport versus multiple sport high school athletes. Int. J. Sports Phys. Ther..

[38] Butler R.J., Lehr M.E., Fink M.L., Kiesel K.B., Plisky P.J. (2013). Dynamic balance performance and noncontact lower extremity injury in college football players: An initial study. Sports Health.

[39] Plisky P.J., Gorman P.P., Butler R.J., Kiesel K.B., Underwood F.B., Elkins B. (2009). The reliability of an instrumented device for measuring components of the star excursion balance test. N. Am. J. Sports Phys. Ther..

[40] Impellizzeri F.M., Marcora S.M., Coutts A.J. (2019). Internal and external training load: 15 years on. Int. J. Sports Physiol. Perform..

[41] McElveen M.T., Riemann B.L., Davies G.J. (2010). Bilateral comparison of propulsion mechanics during single-leg vertical jumping. J. Strength Cond. Res..

[42] Myer G.D., Jayanthi N., DiFiori J.P., Faigenbaum A.D., Kiefer A.W., Logerstedt D., Micheli L.J. (2016). Sports specialization, part II: Alternative solutions to early sport specialization in youth athletes. Sports Health.

[43] Wong P.-L., Chamari K., Chaouachi A., De Wei M., Wisloff U. (2006). Difference in plantar pressure between the preferred and non-preferred feet in four soccer-related movements. Br. J. Sports Med..

[44] de Noronha M., França L.C., Haupenthal A., Nunes G. (2013). Intrinsic predictive factors for ankle sprain in active university students: A prospective study. Scand. J. Med. Sci. Sports.

[45] Iga J., George K., Lees A., Reilly T. (2009). Cross-sectional investigation of indices of isokinetic leg strength in youth soccer players and untrained individuals. Scand. J. Med. Sci. Sports.

[46] González-Fernández F.T., Martínez-Aranda L.M., Falces-Prieto M., Nobari H., Clemente F.M. (2022). Exploring the Y-Balance-Test scores and inter-limb asymmetry in soccer players: Differences between competitive level and field positions. BMC Sports Sci. Med. Rehabil..

[47] Alfredson H., Nordström P., Pietilä T., Lorentzon R. (1998). Long-term loading and regional bone mass of the arm in female volleyball players. Calcif. Tissue Int..

[48] Chu S.K., Jayabalan P., Kibler W.B., Press J. (2016). The kinetic chain revisited: New concepts on throwing mechanics and injury. PmR.

[49] Oliver G.D., Downs J.L., Barbosa G.M., Camargo P.R. (2020). Descriptive profile of shoulder range of motion and strength in youth athletes participating in overhead sports. Int. J. Sports Phys. Ther..

[50] Hori M., Suga T., Terada M., Tanaka T., Kusagawa Y., Otsuka M., Nagano A., Isaka T. (2021). Relationship of the knee extensor strength but not the quadriceps femoris muscularity with sprint performance in sprinters: A reexamination and extension. BMC Sports Sci. Med. Rehabil..

[51] Silva W.A., de Lira C.A.B., Vancini R.L., Andrade M.S. (2018). Hip muscular strength balance is associated with running economy in recreationally-trained endurance runners. PeerJ.

[52] Šuc A., Šarko P., Pleša J., Kozinc Ž. (2022). Resistance exercise for improving running economy and running biomechanics and decreasing running-related injury risk: A narrative review. Sports.

[53] Gokeler A., Welling W., Zaffagnini S., Seil R., Padua D. (2017). Development of a test battery to enhance safe return to sports after anterior cruciate ligament reconstruction. Knee Surg. Sports Traumatol. Arthrosc..

[54] Van Melick N., Van Cingel R.E., Brooijmans F., Neeter C., van Tienen T., Hullegie W., Nijhuis-van der Sanden M.W. (2016). Evidence-based clinical practice update: Practice guidelines for anterior cruciate ligament rehabilitation based on a systematic review and multidisciplinary consensus. Br. J. Sports Med..

[55] Dai B., Layer J., Vertz C., Hinshaw T., Cook R., Li Y., Sha Z. (2019). Baseline assessments of strength and balance performance and bilateral asymmetries in collegiate athletes. J. Strength Cond. Res..

[56] Parkinson A.O., Apps C.L., Morris J.G., Barnett C.T., Lewis M.G. (2021). The calculation, thresholds and reporting of inter-limb strength asymmetry: A systematic review. J. Sports Sci. Med..

